# Editorial: Brain vs. retina - Differences and commonalities: The role of oxidative stress in neurodegenerative diseases

**DOI:** 10.3389/fnins.2023.1171235

**Published:** 2023-03-13

**Authors:** José Hurst, Sven Schnichels

**Affiliations:** Center for Ophthalmology, University Eye Clinic, University Hospital Tübingen, Tübingen, Germany

**Keywords:** neurodegeneration, oxidative stress, retina, ROS, neurological disorder

The articles in this Research Topic shed light on the intricate relationship between oxidative stress and neurodegeneration in ophthalmic and neurological disorders. Oxidative stress is a hallmark of these disorders, and the articles highlight the importance of understanding the mechanisms of oxidative stress in order to develop effective therapeutic interventions. Both the brain and the retina are highly susceptible to oxidative stress, which can contribute to neurodegeneration (Wakamatsu et al., 2008; Angelova and Abramov, 2018; Domanskyi and Parlato, 2022). Oxidative stress is a condition with an imbalance between the production of reactive oxygen species (ROS) and the ability of cells to detoxify or repair the damage caused by ROS (Pizzino et al., 2017). Although the retina is the light sensitive part of the eye, it is also an extension of the brain as it contains central neural tissue and is directly connected to the brain *via* the optic nerve. So, while the retina is technically not a part of the brain, it is directly connected and dependent on the brain for its function in creating our visual perception (Rabin, 2013). Moreover, the exchange of (neurotrophic) factors between the eye and the brain is essential for survival and function of the connected cells, which means, if the counterpart dies, this has direct effects to the corresponding cells in the other organ and subsequently to their up- or downstream connected cells.

In the brain, oxidative stress can be caused by a number of factors, including inflammation, mitochondrial dysfunction and the accumulation of misfolded proteins such as amyloid beta and tau, which are hallmarks of Alzheimer's disease (Korovesis et al., 2023). ROS can damage proteins, lipids as well as DNA, and this damage can lead to neuronal dysfunction and death, which can contribute to the development and progression of neurodegenerative diseases (Pereira et al., 2012; Williams et al., 2013). In addition to Alzheimer's disease, other neurodegenerative diseases such as Parkinson's disease, multiple sclerosis, and Huntington's disease can also manifest in the eye or in its extensions (Archibald et al., 2009; Williams et al., 2013). For example, patients with Parkinson's disease may have visual hallucinations, whereas changes in visual acuity and contrast sensitivity are more common in multiple sclerosis (Zhang et al.).

In the retina, oxidative stress can also be caused by a number of factors, including age-related changes, light exposure and inflammation. The high metabolic rate and constant exposure to light renders the retina particularly vulnerable to oxidative stress, which can generate ROS (Sasaki et al., 2010; Williams et al., 2013). ROS can damage the cells of the retina, including the light-perceiving photoreceptors, and this damage can contribute to the development of age-related macular degeneration and other eye diseases such as glaucoma (Zanon-Moreno and Pinazo-Duran, 2008; Wiktorowska-Owczarek and Nowak, 2010; Marie et al., 2015). However, oxidative stress and inflammation play a role in the development and progression of other retinal diseases such as diabetic retinopathy, retinitis pigmentosa, or Leber's Hereditary Optic Neuropathy (LHON) (Kang and Yang, 2020; Gallenga et al., 2021; Rovcanin et al., 2021).

The article by Ryan et al. explores the role of oxidative stress in traumatic injury to the brain and retina. The study discovered that oxidative stress plays a critical role in neuronal death and tissue damage in the brain and retina after injury (Ryan et al.). Mitochondrial transplantation, as demonstrated in the second article by Lin et al., can mitigate these effects and improve locomotor function after spinal cord injury in rats. Similarly, the article by Zhou et al. reveals that superoxide dismutase 2 can ameliorate mitochondrial dysfunction in skin fibroblasts of patients with LHON, providing a potential therapeutic strategy for this genetic disorder.

In the context of retinal degeneration, the article by Gibbons et al. demonstrates that SARM1 promotes photoreceptor degeneration in an oxidative stress model of retinal degeneration. This finding highlights the importance of targeting SARM1 as a potential therapeutic strategy for retinal degenerative diseases (Gibbons et al.). In addition, the article by Wang et al. shows that complement C3a receptor inactivation can attenuate retinal degeneration induced by oxidative damage, providing further evidence for the role of oxidative stress in retinal degeneration.

The article by Constable et al. provides insights into the electroretinogram (ERG) characteristics of patients with autism spectrum disorder and attention deficit hyperactivity disorder. The study used discrete wavelet transform analysis to identify specific frequency bands that may be associated with these disorders (Constable et al.). Zhang et al. demonstrates a correlation between ophthalmologic problems and cognitive impairment in patients with Parkinson's disease, suggesting ophthalmic examinations may serve as a useful tool for monitoring cognitive decline in these patients. Finally, Wolfrum et al. shed light on the role of p53 in AMD and draw comparisons to the role of p53 in Alzheimer's and Parkinson's disease.

In conclusion, the close relationship between the eye and the brain provides an opportunity for researchers and clinicians to gain insight into the pathogenesis and progression of neurodegenerative diseases and develop novel diagnostic and therapeutic approaches. Collectively, these articles highlight the importance of oxidative stress in the pathogenesis of neurodegenerative and ophthalmic disorders. Future studies should focus on elucidating the mechanisms of oxidative stress and identifying effective therapeutic strategies that can mitigate the damaging effects of oxidative stress on the brain and retina.

Overall, this Research Topic provides a comprehensive overview of the current understanding of the interplay between oxidative stress and neurodegeneration in ophthalmic and neurological disorders. We hope that the findings presented here will inspire further research in this area and ultimately lead to the development of effective treatments for these devastating disorders. Additionally, due to several similarities between eye and brain disorders understanding similar pathomechanisms might lead to dual-use of therapeutics. Moreover, as the retina is visible from the outside, diagnostic techniques to not only diagnose eye diseases but also brain disease should be investigated with a greater extent.

## Author contributions

All authors listed have made a substantial, direct, and intellectual contribution to the work and approved it for publication.
